# Acute Calcific Tendinitis of the Longus Colli Muscle

**DOI:** 10.7759/cureus.50599

**Published:** 2023-12-15

**Authors:** Junki Mizumoto

**Affiliations:** 1 Department of Medical Education Studies, International Research Center for Medical Education, Graduate School of Medicine, University of Tokyo, Tokyo, JPN

**Keywords:** torticollis, neck pain, longus colli muscle, emergency medicine, calcification

## Abstract

A man in his 40s complained of posterior neck pain and headache after a local festival. The patient also developed mild fever, odynophagia, and difficulty opening his mouth widely. Physical examination revealed mild rightward torticollis and limited ranges of neck motion. A neck computed tomography (CT) revealed calcification on the tendon of the lingus colli muscle. The pain decreased rapidly after acetaminophen and loxoprofen administration. Physicians should recognize the clinical characteristics of acute calcific tendinitis of the longus colli muscle (ACTLC) and conduct thorough follow-ups to exclude infection.

## Introduction

To achieve a precise and prompt diagnosis, physicians should meticulously consider the interplay between patient history, thorough physical examination, and diagnostic imaging. This comprehensive approach frequently unveils distinctive and intriguing cases. The presence of odynophagia and posterior neck pain can occasionally signal serious infectious conditions, such as deep neck infection and meningitis. Symptoms associated with soft tissue calcification in the neck may also manifest similarly. While the differential diagnosis can be challenging, the synergistic application of a characteristic patient history, thorough physical examination, and diagnostic imaging can significantly enhance the quality of diagnostic assessments. 

## Case presentation

A previously healthy man at age 42 presented to our emergency department with complaints of posterior neck pain and headache. He reported participating in a local festival two days prior, during which he carried a Mikoshi, or portable shrine, by placing the carrying pole between his right shoulder and the right side of his neck. The neck pain and headache began one day before his presentation. On the morning of the presentation day, he experienced mild fever, pain on the left side of his throat when swallowing, and difficulty opening his mouth widely. Physical examination revealed mild rightward torticollis. While he was able to move his head back and forth with mild to moderate pain, he could not rotate it due to severe pain. There were no signs of redness in the pharynx and tonsils, and no tenderness was noted over the thyroid or jugular veins. There were no swollen or tender lymph nodes.

**Figure 1:**
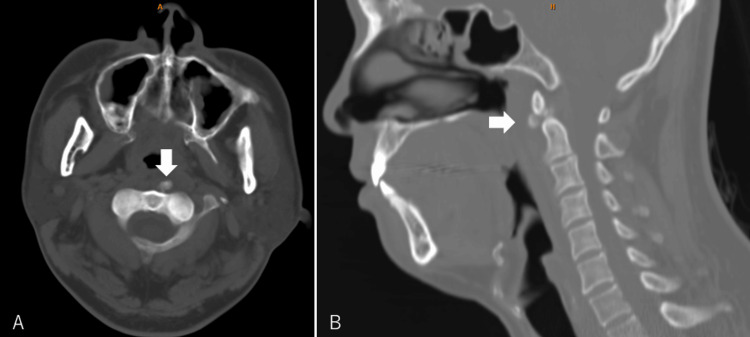
(A) A horizontal view of the neck CT. An arrow indicates the calcification of the longus colli muscle. (B) A sagittal view. An arrow indicates the calcification CT, computed tomography

Considering his history of carrying the Mikoshi, the limited head rotation, and the posterior neck pain accompanied by pain during swallowing, a diagnosis of acute tendinitis of the left longus colli muscle was suspected. A neck computed tomography (CT) revealed calcification on the tendon (Figure 1). He received 1000 mg of intravenous acetaminophen, leading to a rapid decrease in pain. He was prescribed 60 mg tablets of loxoprofen, and the pain subsided within a few days.

## Discussion

Acute calcific tendinitis of the longus colli muscle (ACTLC) is characterized by the deposition of calcium hydroxyapatite and subsequent inflammation in the longus colli muscle, primarily affecting its superior oblique portion [1,2]. Common symptoms include neck pain (experienced by over 90% of patients), limited range of motion (in about half of patients), and neck stiffness (about a half) [3]. ACTLC can impact the retropharyngeal space, leading to symptoms like odynophagia, dysphagia, sore throat, and difficulty in mouth opening [2,3]. Some cases manifest with torticollis [4]. Risk factors include repetitive trauma and recent injuries [5]. The mechanism of developing ACTLC is not fully understood. One hypothesis suggests that trauma, degeneration, or ischemia of the tendon may lead to the deposition of crystals as a compensatory mechanism for reduced tendon quality [6]. ACTLC may go underdiagnosed due to its nonspecific symptoms, self-limiting nature, and lack of familiarity among physicians [5].

Diagnosis is supported by identifying calcification of the tendon anterior to the atlas on CT scans, found in about nine out of 8416 consecutive neck CT scans with no other apparent cause for the patient's symptoms [7]. However, not all cases display calcification [2]. Contrast-enhanced CT showing uniform fluid retention without rim-enhancing effects in the anterior space of the first to sixth cervical vertebrae and an absence of suppurative retropharyngeal lymphadenopathy or other structural abnormalities can substantiate the diagnosis [2,5]. Magnetic resonance imaging (MRI) can identify prevertebral edema and fluid effusion but lacks the capability to detect calcium deposits. Therefore, CT may work better than MRI in diagnosing prevertebral calcification [8]. Differential diagnosis is crucial, especially to distinguish ACTLC from conditions like retropharyngeal abscess and meningitis [5]. In some cases, the diagnosis becomes complex when bacterial infection occurs alongside asymptomatic calcification [9,10]. Given that ACTLC pain swiftly resolves with non-steroidal anti-inflammatory drug administration, close monitoring is essential [3,4]. Physicians should be aware that ACTLC rarely occurs in individuals younger than 20 years old [7].

## Conclusions

A typical history of preceding neck strain, symptoms including odynophagia and limited motion of the neck, and a CT finding of retropharyngeal calcification may indicate ACTLC. It is imperative for physicians to recognize the clinical characteristics of ACTLC and conduct thorough follow-ups, confirming that the pain completely dissipates within a few days, to exclude infection. This approach is essential for ensuring an efficient and accurate diagnosis.
